# Dipeptidyl Peptidase-4 and Adolescent Idiopathic Scoliosis: Expression in Osteoblasts

**DOI:** 10.1038/s41598-017-03310-x

**Published:** 2017-06-09

**Authors:** Emilie Normand, Anita Franco, Alain Moreau, Valérie Marcil

**Affiliations:** 1 Research Center of the Sainte-Justine University Hospital, Montreal, Quebec H3T 1C5 Canada; 2 Department of Nutrition, Faculty of Medicine, Université de Montreal, Montreal, Quebec H3T 1J4 Canada; 3 Viscogliosi Laboratory in Molecular Genetics of Musculoskeletal Diseases, Research Center of the Sainte-Justine University Hospital, Montreal, Quebec H3T 1C5 Canada; 4 Department of Biochemistry and Molecular Medicine, Faculty of Medicine, Université de Montreal, Montreal, Quebec H3T 1J4 Canada; 5 Department of Stomatology, Faculty of Dentistry, Université de Montréal, Montreal, Quebec H3A 1J4 Canada

## Abstract

It has been proposed that girls with adolescent idiopathic scoliosis (AIS) tend to have a taller stature and a lower body mass index. Energy homeostasis, that is known to affect bone growth, could contribute to these characteristics. In circulation, dipeptidyl peptidase-4 (DPP-4) inactivates glucagon-like peptide-1 (GLP-1), an incretin that promotes insulin secretion and sensitivity. Our objectives were to investigate DPP-4 status in plasma and in osteoblasts of AIS subjects and controls and to evaluate the regulatory role of metabolic effectors on DPP-4 expression. DPP-4 activity was assessed in plasma of 113 girls and 62 age-matched controls. Osteoblasts were isolated from bone specimens of AIS patients and controls. Human cells were incubated with glucose, insulin, GLP-1 and butyrate. Gene and protein expressions were evaluated by RT-qPCR and Western blot. Our results showed 14% inferior plasma DPP-4 activity in AIS patients when compared to healthy controls (P = 0.0357). Similarly, osteoblasts derived from AIS subjects had lower DPP-4 gene and protein expression than controls by 90.5% and 57.1% respectively (P < 0.009). DPP-4 expression was regulated in a different manner in osteoblasts isolated from AIS participants compared to controls. Our results suggest a role for incretins in AIS development and severity.

## Introduction

Adolescent idiopathic scoliosis (AIS) is a three dimensional malformation of the spine of unknown cause appearing in children between the age of 10 years old and adulthood^1^. This type of scoliosis affects about 2% of all adolescents and mostly girls, with a ratio of 10 girls to every boy^1, 2^. Patients affected with AIS can have mild (Cobb angle under 25°), moderate (25 to 45°) or severe (≥45°) scoliotic curvature and treatment will differ according to disease severity^1, 2^.

Several studies have shown that girls with AIS tend to have different anthropometric features compared to age-matched controls such as a taller stature, lower body mass index and systemic low bone mass, but the causes of these differences remain unexplained^3–5^. As a matter of fact, comparing nutritional intake of girls affected with AIS and healthy controls showed no significant discrepancies regarding calorie, calcium or protein consumption^6^. While differences in energy homeostasis (appetite regulation, energy expenditure, insulin sensitivity) could contribute to explain these differences, these components have been poorly explored in AIS.

Limited studies have examined the role of leptin in AIS, a hormone produced by the adipose tissue that regulates hunger. Interestingly, leptin can also affect bone metabolism through direct and indirect mechanisms^7–10^. Two studies have shown lower serum leptin levels in girls with AIS than in controls^11, 12^, however others have disputed these findings^13^, leaving the role of leptin in AIS etiopathology and related abnormal bone growth unclear. Though the observations of Liu *et al*. failed to show a difference in serum leptin concentrations, abnormal leptin bioavailability was proposed as a contributing factor to AIS etiology^13^. In addition, a study found no clear association between variations in leptin gene expression and AIS^14^. However, others revealed that a polymorphism in the leptin receptor (*LEPR*) gene was associated with the occurrence of AIS, supporting the idea that malfunctions in the leptin signalling pathway could contribute to AIS development^15^.

Energy homeostasis, that influences bone mass and bone growth^16^, is maintained by the interplay between several hormones^17^. In addition to adipokines secreted by the adipose tissue, the gastro-intestinal (GI) tract secretes peptides in response to nutrient intake^18^. These incretins modulate glucose homeostasis, mainly through glucose-induced insulin secretion, inhibition of glucagon release and enhancement of insulin sensitivity^19^, but accumulating evidence suggests that they may also influence bone metabolism^20, 21^. It was proposed that GI hormones could contribute to reduced bone resorption after a meal^20^. Of particular interest, GI-secreting peptides include glucagon-like peptide-1 (GLP-1), which promotes insulin secretion by interacting with its receptor (GLP-1R) on pancreatic β cells^22, 23^ and enhances insulin sensitivity in peripheral tissues^22^. New evidences indicate that short-chain fatty acids including butyric acid, that are products of non-digestible carbohydrate fermentation by gut microbiota, can stimulate GLP-1 secretion, thereby influencing glucose homeostasis and energy balance^24, 25^ and supporting the role of gut microbiota in bone health^26, 27^.

GLP-1 is secreted by intestinal L cells as a 7–37 or 7–36 amide peptide and is rapidly inactivated in circulation by the aminopeptidase dipeptidyl peptidase-4 (DPP-4), a multifunctional membrane-anchored enzyme mainly produced by endothelial cells^28^. DPP-4 inhibitors, a class of drugs on the market for the treatment of type 2 diabetes^29^, were shown to decrease the risk of bone fractures when compared to standard diabetes treatments (insulin and thiazolidinediones) and placebos^30^, establishing a link between DPP-4 and bone quality. DPP-4, by controlling GLP-1 inactivation, is a tight regulator of energy homeostasis, insulin secretion and sensitivity. Since these phenomena are known to influence bone growth in children and adolescents^31, 32^, it is possible that they contribute to the AIS etiology, progression and/or severity.

In this study, we aimed to investigate DPP-4 status in plasma and in osteoblasts of AIS subjects and controls. Our study also intended to evaluate the regulatory role of metabolic effectors (glucose, insulin, GLP-1 and butyrate) on DPP-4 expression in osteoblasts derived from AIS and control subjects.

## Results

### DPP-4 activity in plasma

**Figure 1:**
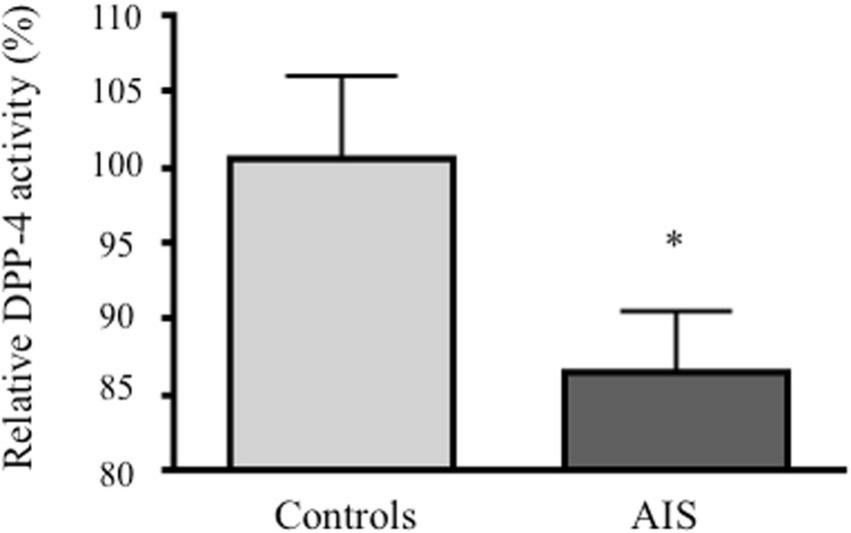
Plasma DPP-4 activity. DPP-4 activity was measured using the DPPIV/CD26 Enzo Life Science’s assay kit in plasma of 113 AIS girls and 62 age-matched controls. *P < 0.05 using two-tailed Student’s t-test. Data are presented as mean ± standard deviation.

Biochemical assays were performed on plasma of girls with AIS and matched controls to assess DPP-4 enzymatic activity. No difference in age was noted between groups, but wide ranges in body mass index and Cobb angles were observed within the AIS cohort (Table 1). As shown in Fig. 1, plasma DPP-4 activity of AIS patients was 14% lower than that of controls (P = 0.0357), suggesting less GLP-1 cleavage in circulation. To verify the effect of body mass index on DPP-4 activity, the AIS cohort was stratified using BMI-for-age percentiles according to the Center for Disease Control and Prevention (CDC, 2016) charts. No significant difference in DPP-4 activity was found between groups (data not shown). Next, we stratified the AIS cohort according to participants’ highest Cobb angle and observed lower DPP-4 activity only in AIS subjects with Cobb angles superior to 30° and the diminution was found statistically significant only in girls with Cobb angles >50° (Supplementary Figure 1).

**Table 1:** Demographic data of subjects tested for plasma DPP-4 activity.

Group	N	Age		BMI		Highest Cobb angle (°)	
		Mean	Range	Mean	Range	Mean	Range
Controls	62	14.3 ± 1.5	11.6–16.5	n/a	n/a	—	—
AIS	113	13.7 ± 1.4	11.6–16.7	19.5 ± 3.7	14.1–34.5	33 ± 15	10–71

DPP-4: Dipeptidyl Peptidase-4; BMI: Body mass index; AIS: Adolescent idiopathic scoliosis.

### DPP-4 expression in osteoblasts

**Figure 2:**
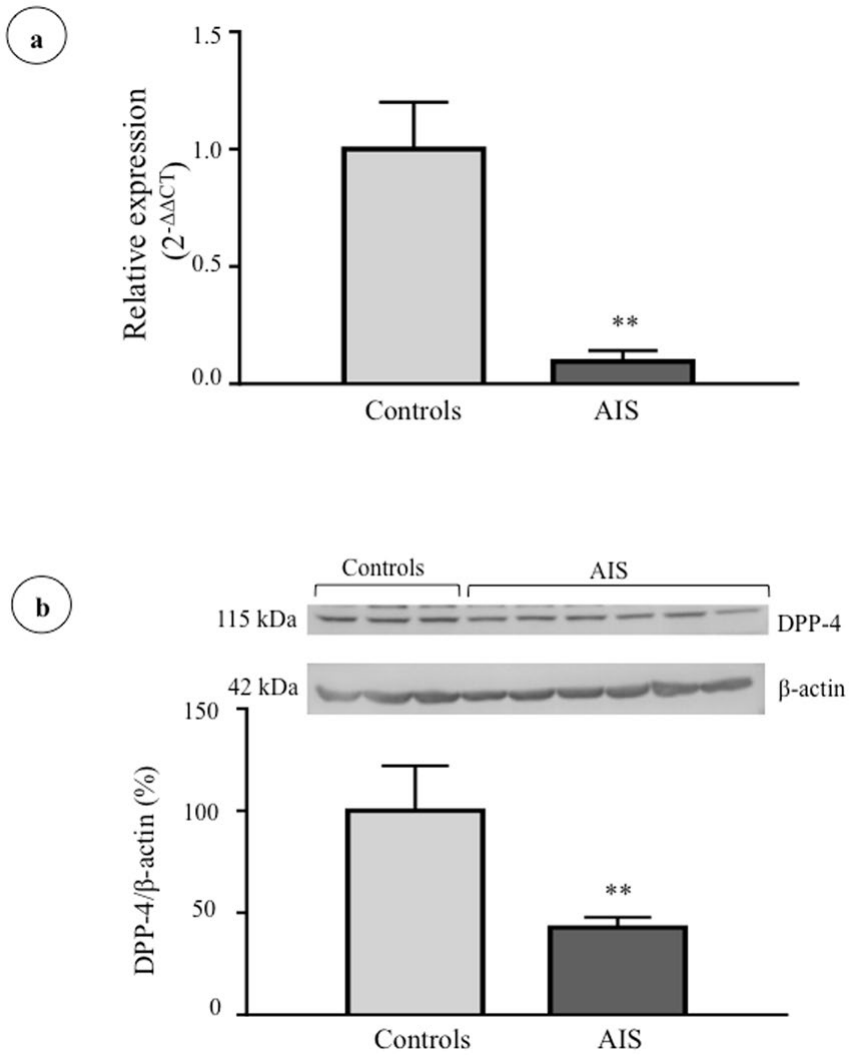
DPP-4 gene and protein expression in osteoblasts isolated from controls and AIS patients. (**a**) *DPP4* gene expression was measured by RT-qPCR with *GAPDH* as endogenous control (n = 7/group). Relative expression was analyzed with the 2^−ΔΔCT^ method. (**b**) DPP-4 protein expression was evaluated by Western blot (n = 3 controls and n = 6 AIS patients) and β-actin was used as endogenous control. **P < 0.01 using two-tailed Student’s t-test. Data are presented as mean ± standard deviation.

With this set of experiments, we aimed to compare total cellular DPP-4 expression in osteoblasts isolated from AIS subjects and healthy controls (cohorts detailed in Supplementary Tables 1 and 2). Our results revealed a marked decrease in DPP-4 gene and protein expression (respective decrease of 90.5% and 57.1%, P < 0.009) in AIS patients compared to controls (Fig. 2). Under our experimental conditions, secreted DPP-4 was undetectable in cell culture media. Osteoblasts from AIS patients were obtained intra-operatively during spine surgery (collected at curve apex), whereas osteoblasts for controls were isolated from leg bones of trauma cases. Therefore, we aimed to validate whether the observed differences in DPP-4 expression were related to the specimen collection site. To do so, we studied DPP-4 expression in osteoblasts derived from spines and femurs of three animal species (mouse, rabbit and rat). Our results showed equivalent levels of DPP-4 gene and protein expression between osteoblasts derived from femurs and spines for all 3 species (Supplementary Figure 2), supporting that the observed differences between AIS and controls were not due to the anatomical sites of bone specimen collection.

### Osteoblast mineralization and cell viability

We used alizarin red staining to examine the degree of mineralization of osteoblasts derived from AIS and control subjects. There was no significant difference observed in the mineralization status between groups after 2, 3 and 4 weeks (Supplementary Figure 3). To verify the integrity of the osteoblasts used in our experiments, cells were examined for the presence of the bone biomarker alkaline phosphatase (ALP). Both groups showed a positive result as demonstrated by the purple color on cellular monolayers (Supplementary Figure 4), thus validating the presence of mature osteoblasts in both groups. In our laboratory, all cultured osteoblasts are routinely tested for bone markers such as *RUNX2, BMP2, PTGS2* and *OPN* using RT-qPCR. We observed little to no difference between cells derived from AIS and control participants as previously described by Oliazadeh *et al*.^33^.

### Modulation of DPP-4 expression by metabolic effectors

**Figure 3:**
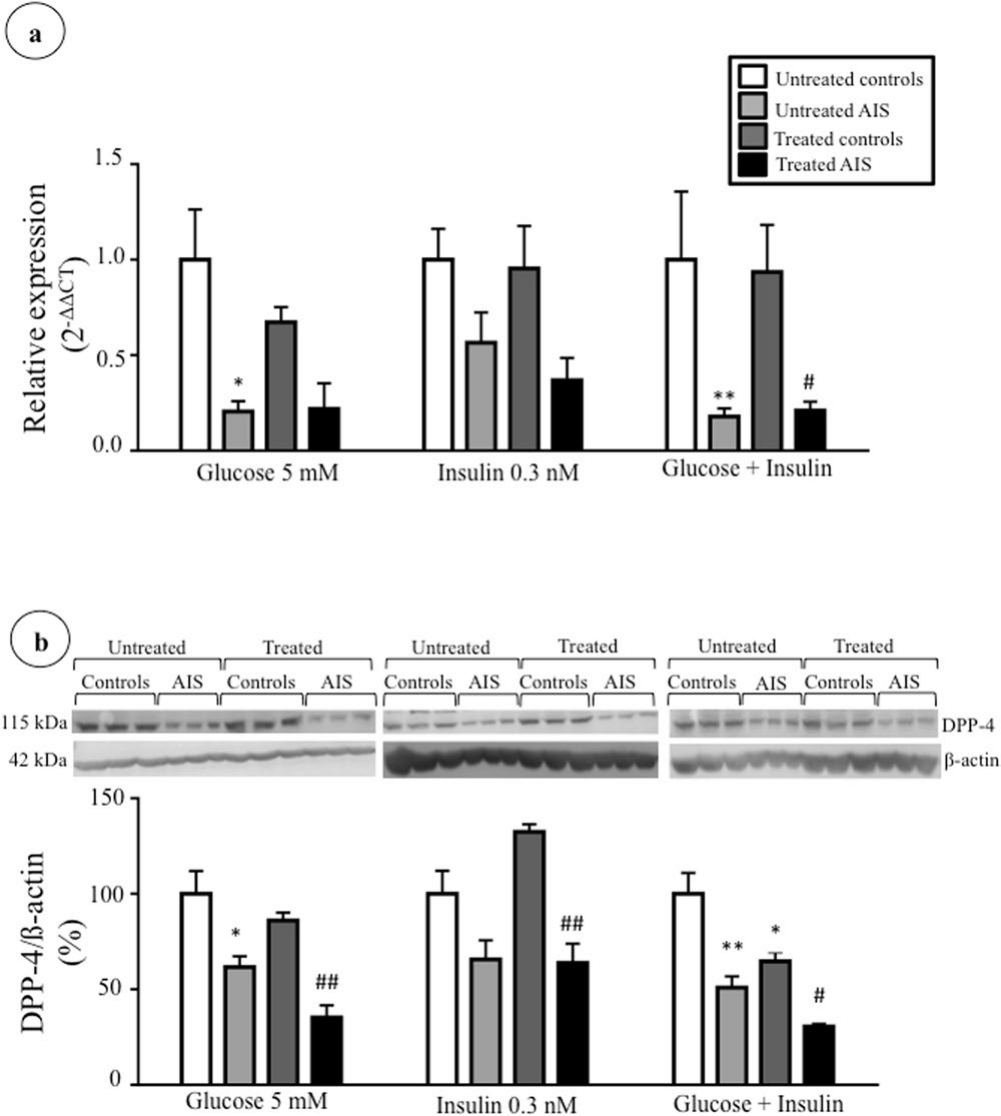
Impact of short-term treatments with glucose and insulin on DPP-4 expression. Cells were treated with glucose (5 mM), insulin (0.3 nM) and glucose + insulin (5 mM and 0.3 nM) for 2 hours. (**a**) *DPP4* gene expression in osteoblasts of controls (n = 3) and AIS patients (n = 4–6) was measured by RT-qPCR with *GAPDH* as endogenous control. Relative expression was analyzed with the 2^−ΔΔCT^ method. (**b**) DPP-4 protein expression in osteoblasts of controls and AIS patients (n = 3/group) was measured by Western blot with β-actin as endogenous control. *P < 0.05 and **P < 0.01 vs. untreated controls; ^#^P < 0.05 and ^##^P < 0.01 vs. treated controls using one-way ANOVA followed by Tukey’s post-hoc tests. Data are presented as mean ± standard deviation.

**Figure 4:**
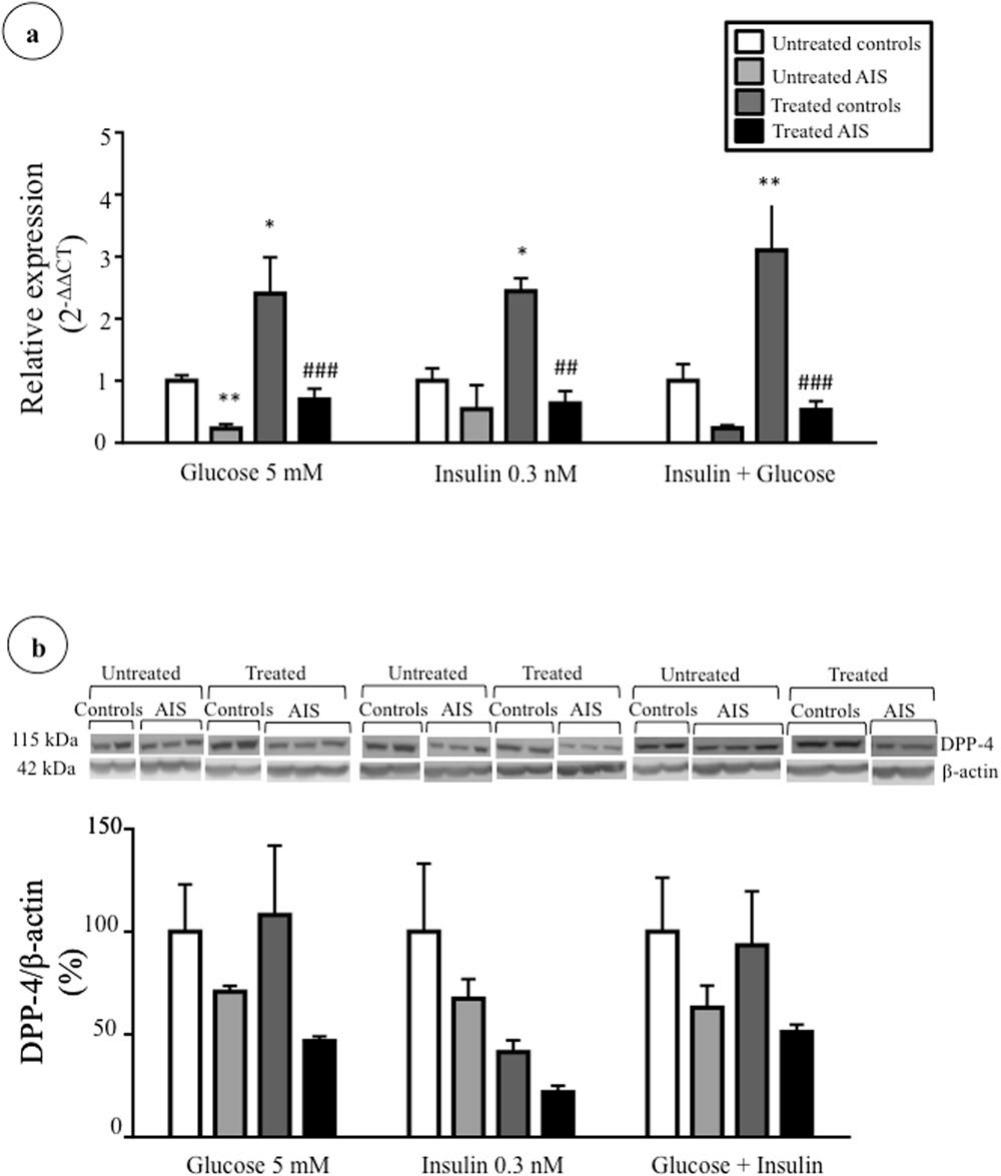
Impact of long-term treatments with glucose and insulin on DPP-4 expression. Cells were treated with glucose (5 mM), insulin (0.3 nM) and glucose + insulin (5 mM and 0.3 nM) for 24 hours. (**a**) *DPP4* gene expression in osteoblasts of controls (n = 3) and AIS patients (n = 4–6) was measured by RT-qPCR with *GAPDH* as endogenous control. Relative expression was analyzed with the 2^−ΔΔCT^ method. (**b**) DPP-4 protein expression in osteoblasts of controls and AIS patients (n = 3/group) was measured by Western blot with β-actin as endogenous control. *P < 0.05 and **P < 0.01 vs. untreated controls; ^##^P < 0.01 and ^###^P < 0.001 vs. treated controls using one-way ANOVA followed by Tukey’s post-hoc tests. Data are presented as mean ± standard deviation.

**Figure 5:**
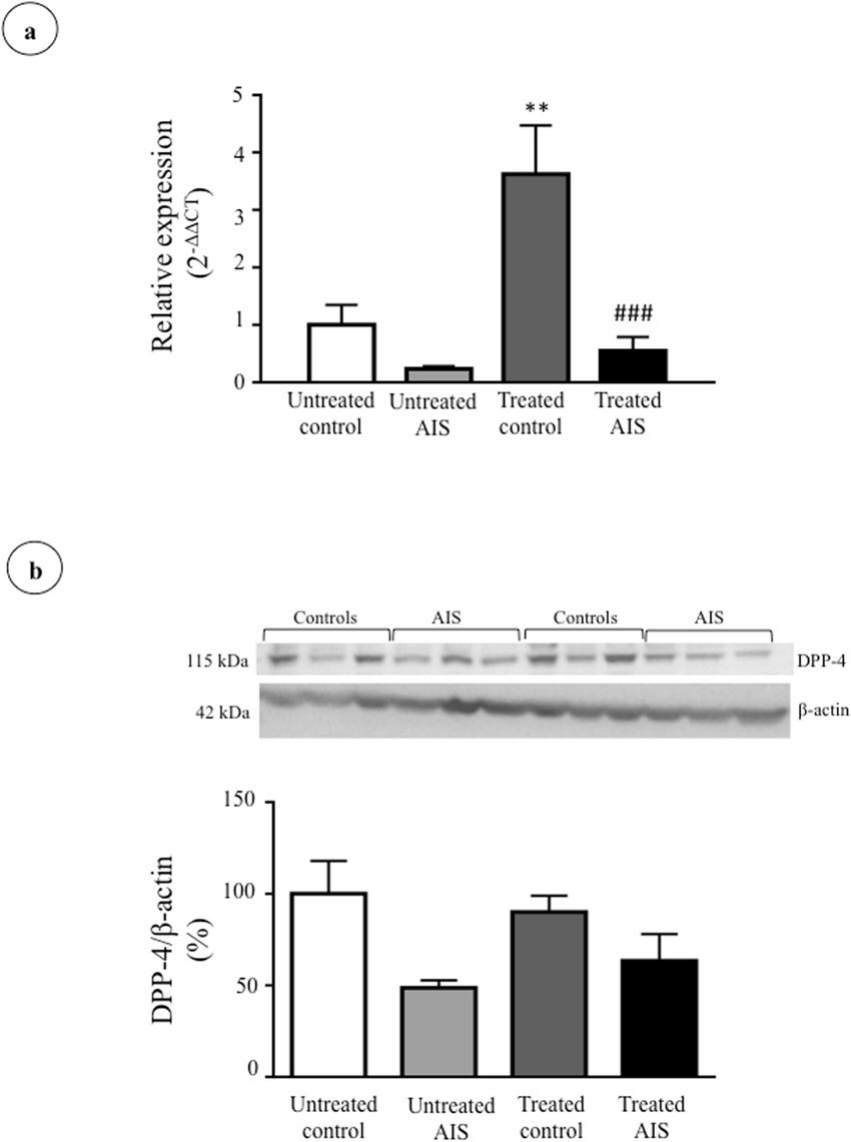
Impact of long-term treatment with GLP-1 on DPP-4 expression. Cells were treated with GLP-1 (10 mM) for 24 hours. (**a**) *DPP4* gene expression was measured in osteoblasts of controls (n = 3) and AIS patients (n = 6) by RT-qPCR with *GAPDH* as endogenous control. Relative expression was analyzed with the 2^−ΔΔCT^ method. (**b**) DPP-4 protein expression in osteoblasts of controls and AIS patients (n = 3/group) was measured by Western blot with β-actin as endogenous control. **P < 0.01 vs. untreated controls; ^###^P < 0.001 vs. treated controls using one-way ANOVA followed by Tukey’s post-hoc tests. Data are presented as mean ± standard deviation.

We then investigated whether short- (2 h) and long-term (24 h) incubations with metabolic effectors could modify osteoblast DPP-4 expression. First, the impact of effectors on cell viability was assessed using the MTT assay. Compared to cells that did not receive treatment, incubations for 2 and 24 h with glucose (5 mM), insulin (0.3 nM), glucose and insulin (5 mM and 0.3 nM), GLP-1 (10 nM) and butyrate (10 mM) did not significantly affect cell viability (Supplementary Figure 5), thus showing that these effectors were not toxic for the cell cultures. Subsequently, we measured their impact on DPP-4 gene and protein expression using RT-qPCR and Western blot. Results show that most short-term treatments with glucose or insulin did not impact DPP-4 gene and protein expression in a significant manner for control and AIS osteoblasts (Fig. 3a and b). Only incubations in the presence of both glucose and insulin significantly reduced DPP-4 protein expression in control osteoblasts (35% decrease, P < 0.05) without modulating mRNA levels, suggesting a post-transcriptional regulation. Long-term incubations with glucose and/or insulin led to higher *DPP4* gene expression in osteoblasts obtained from control subjects (glucose: 240%, P < 0.05; insulin: 244%, P < 0.05; glucose and insulin: 310%, P < 0.01) (Fig. 4a). However, these increases were not reflected in protein expression (Fig. 4b). Moreover, the impact of short- and long-term incubations with GLP-1 and butyrate were tested. While 2 h incubations with GLP-1 did not impact DPP-4 expression (Supplementary Figure 6), we found that the 24 h treatment had an effect on gene expression in cells obtained from controls (increase of 362% P < 0.01) without affecting protein levels (Fig. 5). Finally, incubations with butyrate did not significantly modulate DPP-4 gene and protein expression (Supplementary Figures 7 and 8). Of note, at both time points and with all effectors, the expression of DPP-4 in AIS osteoblasts was inferior to controls.

### STAT1 expression in osteoblasts

**Figure 6:**
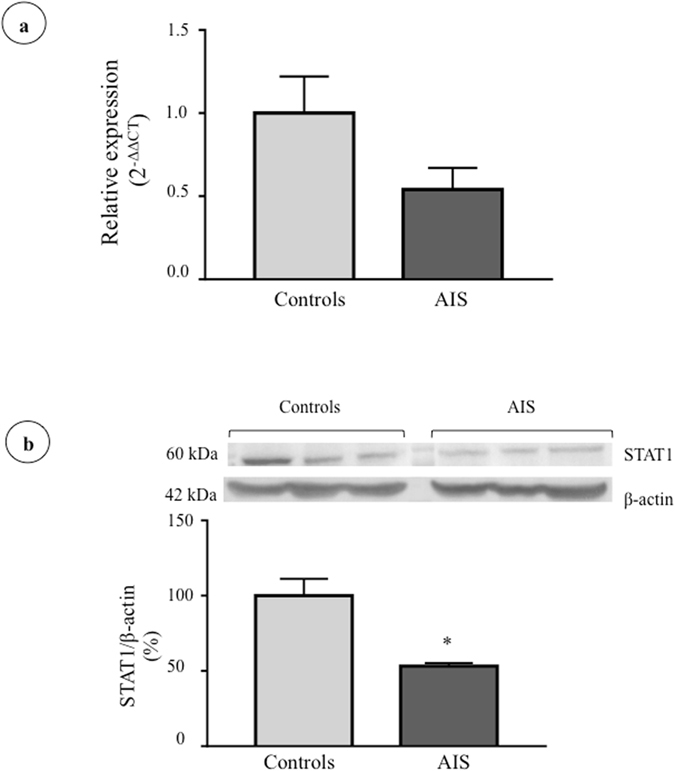
STAT1 gene and protein expression in osteoblasts isolated from control and AIS patients. (**a**) *STAT1* gene expression was measured in osteoblasts of controls (n = 7) and AIS patients (n = 13) by RT-qPCR with *GAPDH* as endogenous control. Relative expression was analyzed with the 2^−ΔΔCT^ method. (**b**) STAT-1 protein expression was evaluated by Western blot (n = 3/group) and β-actin was used as endogenous control. *P < 0.05 using two-tailed Student’s t-test. Data are presented as mean ± standard deviation.

Finally, we compared gene and protein expression of the Signal transducer and activator of transcription 1 (STAT1), a known DPP-4 transcriptional regulator, in osteoblasts derived from control and AIS subjects (cohorts detailed in Supplementary Tables 1 and 2). Our results revealed a 46% reduction in STAT1 gene expression, which was not statistically significant (Fig. 6a) and a similar lower protein expression in AIS cells (47% reduction, P < 0.015) (Fig. 6b).

## Discussion

In this study, we found lower DPP-4 activity in plasma of AIS girls compared to age-matched female controls. Stratification by higher Cobb angle showed that DPP-4 activity decreased with curve severity, that this decline became apparent at the 30° mark and was significant for curves above 50°. We also confirmed that DPP-4 is expressed in osteoblasts and that DPP-4 gene and protein expression is lower in osteoblasts of AIS patients compared to controls. Results obtained in animal studies validate our findings of observed differences to be in fact related to the disease rather than the collection sites. We also found that, in AIS osteoblasts, DPP-4 expression is regulated by glucose, insulin and GLP-1 in a different manner than in cells obtained from controls. These results support the hypothesis of a relation between insulin sensitivity and AIS. Accordingly, other groups have found that patients with AIS have increased fasting total ghrelin levels, a peptide hormone that stimulates food intake^34^, possibly in part by modulating the effect of GLP-1^35^. Authors put forward that ghrelin signalling could play a role in AIS development and explain lower body weight and body mass index in AIS girls and also suggested a role of GI hormones in AIS physiopathology.

The optimal regulation of GLP-1 actions is based on its partial degradation by the enzyme DPP-4^28^. Therefore, it is possible that in AIS, lower levels of DPP-4 result in less cleavage and higher active GLP-1 in circulation. Active GLP-1 binds the GLP-1R on pancreatic β cells, stimulating insulin secretion and thus plays a role in bone growth^22, 31^. Insulin can influence bone cell activity by binding to the insulin receptor on osteoblasts^36^. Given the role of DPP-4 in energy homeostasis, we hypothesize that enhanced insulin response and sensitivity could contribute to explain abnormal bone growth of patients during puberty. This may possibly be linked to the severity and/or progression of AIS and also explain in part the different anthropometric features reported, such as lower body mass index^3^.

DPP-4 inhibitors, used as antidiabetic drugs, are known to decrease bone fracture compared to standard diabetes treatment and placebo^29^. Conversely, Cheng *et al*. reported osteopenia and early onset osteoporosis in AIS patients^37^. However, this study was done exclusively in a population from Hong Kong and bone health has not been well characterized in Caucasian cohorts. Therefore, further data are needed to determine the impact of lower DPP-4 activity on bone features of AIS patients.

We focused our experiments on DPP-4 instead of GLP-1 directly, mainly because that in order to correctly assess the active form of GLP-1, it is required to treat the blood with a DPP-4 inhibitor within 30 seconds of veinipuncture^38^. In our case, this procedure was not possible as the plasma samples were obtained from an archived biobank. Moreover, because the fasting state of subjects was unknown, measuring GLP-1 levels could possibly lack accuracy^39^. However, studies have reported that plasma DPP-4 activity was not different between fasting and post-prandial states^40, 41^. Other possible confounding factors of DPP-4 activity include participants’ body mass index, although no differences were found in our cohort, bone mineral density and gender. According to the literature, the correlation between bone mineral density and DPP-4 activity varies according to the population studied. Recently, Carbone L.D. *et al*.^42^ reported no association between plasma DPP-4 activity and bone mineral density in a cohort of 1536 elderly adults. Another study performed in obese postmenopausal women found that low bone mineral density was positively correlated with serum DPP-4 activity^43^. Also, Carbone L.D. *et al*. found no difference in plasma DPP-4 activity between male and female participants^42^.

For all treatments and time points we observed lower DPP-4 expression in AIS osteoblasts compared to controls, indicating that metabolic effectors do not influence this disparity. This might reflect a concomitant lesser production by other tissues and could contribute to inferior levels of DPP-4 in plasma of AIS subjects. DPP-4 is widely expressed in the human body, as it is found in many tissues and cells, but mostly on the surface of endothelial cells including blood vessels passing through the GI tract^44^. However, the contribution of the bone to circulating DPP-4 remains unknown. There is very little literature exposing DPP-4 in relation to osteoblasts and this is surely a field that needs more investigation. While secreted DPP-4 could not be detected under our experimental conditions, Das *et al*.^73^ successfully studied released DPP-4 from adipocytes, which could indicate higher secretion from adipocytes than osteoblasts. The same authors also showed that adipocytes cultured with a high glucose concentration (25 mM) expressed less intracellular DPP-4 than cells incubated with lower concentrations (5 mM)^73^. Besides, Kyle *et al*. studied in mice the effects of a DPP-4 chemical reduction using a DPP-4 inhibitor on bone quality and the impact of *Dpp4* genetic inactivation^45^. Their results showed that female mice treated with the DPP-4 inhibitor had better vertebral volumetric bone mineral density and trabecular architecture. However, *Dpp4−/−* mice did not present any significant difference in their bone features. It was proposed that a total inactivation of the gene could lead to compensatory changes hiding an impact on bone, compared to a partial inactivation of activity with a DPP-4 inhibitor^45^. Moreover, using mice that do not express the insulin receptor on their osteoblasts, the need for insulin for optimal bone development was demonstrated^36^.

There is limited knowledge on the molecular mechanisms that regulate DPP-4 enzymatic activity and gene transcription and they possibly vary according to cell type^46^. The cytosine and guanine rich region in the *DPP4* gene promoter is a known site for the binding of transcription factors involved in the regulation of metabolic pathways^47^. *DPP4* promoter also contains a gamma interferon activation site (GAS), a sequence to which the transcription factor STAT-1 binds. The binding of STAT-1 to the GAS region is stimulated by interferons α, β and γ and it is known to up-regulate the expression of cell-surface and intracellular DPP-4 as well as DPP-4 activity^48^. A study showed a 42% increase of functional DPP-4 activity following a 48 h-incubation with interferon α on chronic B lymphocytic leukemia cells^49^. Our results support the hypothesis that a reduction in STAT-1 expression could be a plausible mechanism explaining lower DPP-4 in AIS osteoblasts.

In our study, osteoblasts were treated with different metabolic effectors that are implicated, directly or indirectly, in the DPP-4 pathway to evaluate whether they modulate DPP-4 gene and protein expression. While incubations with glucose at normal physiological concentrations^50–52^ did not modify DPP-4 expression, long term incubations with insulin (corresponding to physiological concentrations of post-prandial state^53^), elevated DPP-4 gene expression in osteoblasts from controls while reduced protein expression. This indicates possible post-transcriptional regulation. For instance, DPP-4 can be regulated by post-transcriptional mechanisms like methylation^54^, glycosylation^55^ and phosphorylation^56^. Together, our results indicate that DPP-4 expression can be regulated in a different manner in AIS versus control cells.

A newcomer in studies vis-à-vis regulation of metabolic pathways is the gut microbiota^57^. As a matter of fact, disorders like obesity and type 2 diabetes have been linked with variations in gut microbiota composition^58–60^. Evidence also suggest that short-chain fatty acids (acetate, propionate and butyrate), products of non-digestible carbohydrate by gut microbiota fermentation, can stimulate the secretion of incretins such as GLP-1.

Studies in animals^61^ and in humans^24^ have confirmed the link between gut microbiota fermentation and production of GLP-1. A relationship between gut microbiota and bone health is also supported: butyrate is now considered a treatment for bone loss because of its action as a histone deacetylase inhibitor which regulates osteoblast differentiation and bone formation^26, 27^. Moreover, studies have shown that prebiotics could be used to prevent osteoporosis^62, 63^ and in HepG2 cells, butyrate induced higher DPP-4 activity^46^. However, in our study, a treatment with 10 mM butyrate^64, 65^ did not impact DPP-4 expression in osteoblasts. It has been documented that butyrate stimulates incretin secretion by binding to free fatty acid receptors (FFAR1 and FFAR2)^66^ that are G protein-coupled receptors^67^. A study by Akoumé *et al*. reported a signalling dysfunction of G inhibitory proteins in AIS patients^68^, which could explain why treatments with butyrate showed no variation in DPP-4 expression.

GLP-1 at a physiological concentration (10 nM)^69, 70^ only influenced DPP-4 expression in control osteoblasts. It is possible that GLP-1 signalling in AIS patients could be impaired given the occurrence of G inhibitory proteins malfunction in AIS^68^ and the role of cyclic adenosine monophosphatase (cAMP) as a key mediator of the GLP-1 pathway^71^. To this day, GLP-1R expression in osteoblasts has not been clearly confirmed^72^. Although GLP-1 plays a role in bone modeling and bone growth, the mechanisms explaining these functions are still unclear. Treating hindlimb-unloading rats with exendin-4, a GLP-1R agonist, promoted bone formation, bone mass and bone quality^72^. In this study, GLP-1R was not found expressed in osteoblasts, but was detected in bone marrow stromal cells. Also, it was revealed that the activation of GLP-1R on bone marrow stromal cells can indirectly promote osteoblast differentiation through the regulation of PKA/β-catenin and PKA/PI3K/AKT/GSK3β pathways^72^.

A first limitation of our study is that some clinical and demographic data that could consist in confounding factors were unavailable, such as participants’ nutritional status, level of physical activity and bone mineral status. Another limitation is that, because plasma and bone specimen were not collected from the same participants, DPP-4 activity and expression could not be compared. Moreover, since bone specimens were collected intraoperatively, the osteoblasts used for our cellular experiments originated only from severe scoliosis cases.

In conclusion, our study revealed differences in DPP-4 activity, expression levels and regulation between AIS girls and healthy controls. Additional studies are needed to further confirm the role of DPP-4 and incretins in AIS development and severity.

## Methods

### Population

AIS subjects were examined by an orthopedic surgeon at Sainte-Justine University Hospital in Montreal, Canada. Subjects were deemed to be affected if history and physical examination were consistent with the diagnosis of idiopathic scoliosis and a minimum of a ten-degree curvature in the coronal plane with vertebral rotation was found by X-Ray imaging. Healthy controls for plasma collection were recruited in elementary and high schools in the larger Montreal area. Bone specimens were collected intraoperatively from vertebras (varying from T3 to L4 according to the surgical procedure performed) and from tibias or femurs, in AIS patients and trauma cases respectively. Osteoblasts used for cellular biology experiments were derived from female subjects, except for unstimulated *DPP4* gene expression, for which samples from 4 male subjects were added. Approval was obtained by the Institutional Review Boards of Sainte-Justine Hospital, Montreal Children’s Hospital, The Shriners Hospital for Children in Montreal, The Affluent School Board and The Montreal English School Board, in accordance to respective guidelines. Parents or legal guardians of all participants gave written informed consent, and minors also gave their assent. All experimental protocols were approved by the Institutional Review Boards of Sainte-Justine Hospital.

### DPP-4 plasma activity

Peripheral blood samples of AIS girls (n = 113) and age-matched female controls (n = 62) were collected in EDTA-treated tubes and centrifuged. Derived plasma samples were aliquoted and kept frozen at −80 °C until thawed and analyzed. DPP-4 activity in plasma was measured using the DPPIV/CD26 Enzo Life Science’s assay kit which allows for the measurement of DPP-4 activity (Farmingdale, NY, USA), following the manufacturer’s protocol. The activity was expressed in units of µmol/min/L (U/L), with 1 U representing amount of substrate H-gly-pro-pNA hydrolysed per minute. Data were then calculated as percentage of DPP-4 activity in controls.

### Human cell preparation and culture

Human osteoblasts were isolated from bone specimens obtained intraoperatively as previously described^74^. Each biopsy specimen was carefully cleared of remaining fatty and connective tissue prior to being cut into smaller pieces that were repeatedly rinsed in PBS 1X and centrifuged. Bone fragments were cultured in α-MEM culture media (Wisent Bio Products, St-Bruno, QC, Canada) supplemented with 10% fetal bovine serum (FBS, Hyclone Laboratories, Logan, UT, USA) and 1% antibiotic/antimycotic (Thermo Fisher Scientific, Waltham, MA, USA). Cultures were allowed to grow to confluence at 37 °C, 5% CO_2_.

### Animal cell preparation and culture

Bone specimens of untreated wild-type mice, rats and rabbits were collected at the Sainte-Justine Hospital animal facility. The spines and femurs of 6 mice (species: C57Bl/6, all male), 2 rabbits (species: New-Zealand, both male) and 5 rats (species: Sprague Dawley, unknown genders) were collected post-euthanasia. Specimens were processed and cultured as described above. All experiments were performed in accordance with relevant guidelines and regulations and were approved by Sainte-Justine Hospital Institutional Committee of Good Animal Practice in Research.

### Cell treatments

The regulation of DPP-4 expression by metabolic effectors was evaluated by incubating human osteoblasts with five different compounds and/or combination of compounds for 2 and 24 h. Effectors used were glucose (5 mM, Sigma-Aldrich, Oakville, ON, Canada), insulin (0.3 nM, Humulin R, Eli Lilly, Indianapolis, IN, USA), glucose + insulin (5 mM and 0.3 nM), GLP-1 (10 nM, Sigma-Aldrich) and butyric acid (10 mM, Sigma-Aldrich). At confluence, after a 16 h pre-incubation with serum-free α-MEM media, effectors were mixed into serum-free α-MEM media before being added to the wells and incubated at 37 °C. For each sample and time point, the negative control consisted of cells incubated only with serum-free α-MEM media.

### Quantitative real-time PCR

Total RNA was isolated from confluent osteoblasts seeded at a concentration of 60,000 cells/mL, using TRIzol reagent (Thermo Fisher Scientific) according to the manufacturer’s protocol. RNA (500 ng) was reverse-transcribed into cDNA using All-In-One RT MasterMix (Applied Biomedical Materials, Richmond, BC, Canada). Following cDNA synthesis, qPCR was performed using Taqman gene expression probes for human (DPP4 #*Hs00175210_m1;* GAPDH *#Hs02758991_G1*) and mouse (*Dpp4* #*Mm00494549_m1*; *Gapdh* #*Mm99999915_g1*) according to the manufacturer’s instructions. For rats and rabbits, primers were designed using the Basic Local Alignment Search Tool program by NCBI and synthetized by BioCorp (Montreal, QC, Canada). Primers were: rat *Dpp4*: 5′-aaagagctgagagtcctggag-3′ (forward), 5′-tgagtcatagtcctcccaccg-3′ (reverse); rat *Gapdh*: 5′-ggcaagttcaacggcacagt-3′ (forward), 5′-tggtgaagacgccagtagactc-3′ (reverse); rabbit *dpp4*: 5′-tgagtatctccacagacaagaaga-3′ (forward), 5′-ttctaagagaagaaacagtctatcagg-3′ (reverse); rabbit *Actb*: 5′-ccaaccgcgagaagatga-3′ (forward), 5′-ccagaggcgtacagggatag-3′ (reverse). For rats and rabbits, qPCR was performed using Fast SYBR Green (Thermo Fisher Scientific). Transcript expression was assessed with the 7500 Fast Real-Time PCR System (Applied Biosystems, Foster City, CA, USA), and calculations were performed with the 2^−ΔΔCT^ method using GAPDH or β-actin as internal controls.

### Western blot analysis

For each sample, 2–3 10 cm petri dishes of confluent cells were used for protein extraction. Osteoblasts were homogenized using M-Per (Thermo Fisher Scientific) supplemented with 1X protease inhibitor cocktail (Hoffman- La Roche Limited, Mississauga, ON, Canada) (20 µl/dish) and adequately prepared for Western blotting as previously described^75^. The Bradford assay was used to estimate protein concentration. Loading buffer (6X) was added to protein extracts (30–40 µg) and boiled at 100 °C for 5 minutes, before being separated on a 10% SDS-PAGE gel and blotted onto a nitrocellulose membrane. Primary antibodies were: DPP-4 (ab28340, Abcam, Cambridge, UK) (1:500 and 1:1000 for 10- and 15-well gels, respectively); STAT1 (MA1–037X, Thermo Fisher Scientific) (1:500) and; β-actin (A5441, Sigma-Aldrich) (1:5000 and 1:20000 for 10 and 15-well gels, respectively). Secondary antibodies were DPP-4 (goat anti-rabbit, 31460, Thermo Fisher Scientific) (1:1000 and 1:5000 for 10 and 15-well gels, respectively); STAT1 (goat anti-mouse, 115-035-003, Jackson Immunoresearch, West Grove, PA, USA) (1:1000) and; β-actin (goat anti-mouse, 115-035-003, Jackson Immunoresearch, West Grove, PA, USA) (1:5000 and 1:20000 for 10 and 15-well gels, respectively). Bands were visualized with the SuperSignal Chemiluminescent Substrate (Thermo Fisher Scientific) and data was analysed using the densitometry tool in ImageJ2 software (National Institutes of Health).

### Mineralization assay by alazarin red staining

Osteoblasts from AIS and trauma patients (controls) were cultured to passage 2 or 3 and grown to confluence as described above. Media was replaced by a mineralization-inducing media constituted of complete media with 50 µg/ml ascorbic acid (Sigma-Aldrich) and 10 mM β-glycerophosphate (Sigma-Aldrich). Media was changed 3 times per week. On days 14, 21 and 28 from date of plating, cells were fixed and stained. Cells were rinsed in PBS 1X and fixed in 4% paraformaldehyde (PFA, Thermo Fisher Scientific) and incubated at room temperature for 15 min. Cells were then rinsed in ddH_2_O and incubated with alizarin red solution (40 mM, pH 4.1, Sigma-Aldrich) for 20 min at room temperature. Wells were once again rinsed with ddH_2_O and images were recorded by a HP Scanjet G3110 Photo Scanner (Hewlett-Packard, Palo Alto, Ca, USA). For relative quantification, 10% acetic acid (Thermo Fisher Scientific) was added to each well and incubated at room temperature on a shaker for 30 min. The cellular monolayer was collected, vortexed and incubated at 85 °C for 10 min. Samples were transferred on ice for 5 min and centrifuged at 20,000 *g* for 15 min. The supernatant was neutralized with 2% ammonium hydroxide (Thermo Fisher Scientific) at 2:1 volume. Samples were plated in 96-well plates and optical densities were read at 405 nm using the DTX880 Multimode Detector (Beckman Coulter, Brea, CA, USA). Relative quantification was evaluated by comparing readings of AIS to controls.

### Alkaline phosphatase staining

Cells were plated and cultured as described above. After 2 weeks, they were fixed for staining by incubation with PFA for 2 min and rinsed 3 times with ddH_2_O. 5-Bromo-4-chloro-3-indolyl phosphate/nitroblue tetrazolium (BCIP/NBT) liquid substrate system (Sigma-Aldrich) was added and cells were incubated at 37°C for 10 min. Wells were rinsed twice in PBS 1X and images were collected by photo scanner.

### MTT viability assay

Osteoblasts were seeded in 96-well plates (100,000 cells/well) and after 1 week, cells were incubated with metabolic effectors as described. After treatment, media was replaced with MTT solution (12 mM) (Sigma-Aldrich) prepared in PBS 1X and microplates were incubated at 37 °C, 5% for 2 h. MTT solution was removed and 50 μl DMSO (Sigma-Aldrich) was added. After a 10 min incubation at 37 °C and vigorous mixing, absorbance was read at 540 nm. Percentage of cell viability was evaluated for each treatment and patient by calculating the absorbance ratio to the respective untreated samples. For validation, the experiment was repeated in 48-well plates (50,000 cells/well)..

### Statistics

We performed multiple and single comparisons of means using two-tailed Student-t tests and one-way ANOVA followed by Tukey’s post-hoc-tests. Equality of variance among groups was determined using the F-test of equality of variance. Analyses were achieved using the GraphPad Prism 7.0 software (GraphPad Software, La Jolla, CA, USA). Data are expressed as mean values ± SEM. P value < 0.05 was considered significant.

## Electronic supplementary material

Supplementary data

## References

[1] Hresko MT (2013). Clinical practice. Idiopathic scoliosis in adolescents. N Engl J Med.

[2] Altaf F, Gibson A, Dannawi Z, Noordeen H (2013). Adolescent idiopathic scoliosis. BMJ.

[3] Barrios C (2011). Anthropometry and body composition profile of girls with nonsurgically treated adolescent idiopathic scoliosis. Spine (Phila Pa 1976).

[4] Sadat-Ali M, Al-Othman A, Bubshait D, Al-Dakheel D (2008). Does scoliosis causes low bone mass?A comparative study between siblings. Eur Spine J.

[5] Siu King Cheung C (2003). Abnormal peri-pubertal anthropometric measurements and growth pattern in adolescent idiopathic scoliosis: a study of 598 patients. Spine (Phila Pa 1976).

[6] Lee WT (2005). Generalized low bone mass of girls with adolescent idiopathic scoliosis is related to inadequate calcium intake and weight bearing physical activity in peripubertal period. Osteoporos Int.

[7] Tsuji K, Maeda T, Kawane T, Matsunuma A, Horiuchi N (2010). Leptin stimulates fibroblast growth factor 23 expression in bone and suppresses renal 1alpha,25-dihydroxyvitamin D3 synthesis in leptin-deficient mice. J Bone Miner Res.

[8] Turner RT (2013). Peripheral leptin regulates bone formation. J Bone Miner Res.

[9] Ferron M, Lacombe J (2014). Regulation of energy metabolism by the skeleton: osteocalcin and beyond. Arch Biochem Biophys.

[10] Yadav VK (2009). A serotonin-dependent mechanism explains the leptin regulation of bone mass, appetite, and energy expenditure. Cell.

[11] Qiu Y (2007). Decreased circulating leptin level and its association with body and bone mass in girls with adolescent idiopathic scoliosis. Spine (Phila Pa 1976).

[12] Tam EM (2014). Are volumetric bone mineral density and bone micro-architecture associated with leptin and soluble leptin receptor levels in adolescent idiopathic scoliosis?–A case-control study. PLoS One.

[13] Liu Z (2012). Abnormal leptin bioavailability in adolescent idiopathic scoliosis: an important new finding. Spine (Phila Pa 1976).

[14] Liang G (2012). Normal leptin expression, lower adipogenic ability, decreased leptin receptor and hyposensitivity to Leptin in Adolescent Idiopathic Scoliosis. PLoS One.

[15] Liu Z (2015). Polymorphism of rs2767485 in Leptin Receptor Gene is Associated With the Occurrence of Adolescent Idiopathic Scoliosis. Spine (Phila Pa 1976).

[16] Khor EC, Wee NK, Baldock PA (2013). Influence of hormonal appetite and energy regulators on bone. Curr Osteoporos Rep.

[17] Murphy KG, Bloom SR (2006). Gut hormones and the regulation of energy homeostasis. Nature.

[18] Strader AD, Woods SC (2005). Gastrointestinal hormones and food intake. Gastroenterology.

[19] Holst JJ, Deacon CF, Vilsboll T, Krarup T, Madsbad S (2008). Glucagon-like peptide-1, glucose homeostasis and diabetes. Trends Mol Med.

[20] Mabilleau G, Mieczkowska A, Irwin N, Flatt PR, Chappard D (2013). Optimal bone mechanical and material properties require a functional glucagon-like peptide-1 receptor. J Endocrinol.

[21] Clowes JA, Khosla S, Eastell R (2005). Potential role of pancreatic and enteric hormones in regulating bone turnover. J Bone Miner Res.

[22] Preitner F (2004). Gluco-incretins control insulin secretion at multiple levels as revealed in mice lacking GLP-1 and GIP receptors. J Clin Invest.

[23] Thorens B (1992). Expression cloning of the pancreatic beta cell receptor for the gluco-incretin hormone glucagon-like peptide 1. Proc Natl Acad Sci USA.

[24] Ropert A (1996). Colonic fermentation and proximal gastric tone in humans. Gastroenterology.

[25] Cani PD, Everard A, Duparc T (2013). Gut microbiota, enteroendocrine functions and metabolism. Curr Opin Pharmacol.

[26] Katono T (2008). Sodium butyrate stimulates mineralized nodule formation and osteoprotegerin expression by human osteoblasts. Arch Oral Biol.

[27] Schroeder TM, Westendorf JJ (2005). Histone deacetylase inhibitors promote osteoblast maturation. J Bone Miner Res.

[28] Baggio LL, Drucker DJ (2007). Biology of incretins: GLP-1 and GIP. Gastroenterology.

[29] Meier C, Schwartz AV, Egger A, Lecka-Czernik B (2016). Effects of diabetes drugs on the skeleton. Bone.

[30] Yamagishi S (2012). Comment on: Monami et al. Dipeptidyl peptidase-4 inhibitors and bone fractures: a meta-analysis of randomized clinical trials. Diabetes Care 2011;34:2474-2476. Diabetes Care.

[31] Pollock NK (2011). Adolescent obesity, bone mass, and cardiometabolic risk factors. J Pediatr.

[32] Pollock NK (2010). Lower bone mass in prepubertal overweight children with prediabetes. J Bone Miner Res.

[33] Oliazadeh N, Gorman KF, Eveleigh R, Bourque G, Moreau A (2017). Identification of Elongated Primary Cilia with Impaired Mechanotransduction in Idiopathic Scoliosis Patients. Sci Rep.

[34] Sales de Gauzy J (2015). Fasting total ghrelin levels are increased in patients with adolescent idiopathic scoliosis. Scoliosis.

[35] Chelikani PK, Haver AC, Reidelberger RD (2006). Ghrelin attenuates the inhibitory effects of glucagon-like peptide-1 and peptide YY(3-36) on food intake and gastric emptying in rats. Diabetes.

[36] Fulzele K (2010). Insulin receptor signaling in osteoblasts regulates postnatal bone acquisition and body composition. Cell.

[37] Cheng JC, Tang SP, Guo X, Chan CW, Qin L (2001). Osteopenia in adolescent idiopathic scoliosis: a histomorphometric study. Spine (Phila Pa 1976).

[38] Bak MJ (2014). Specificity and sensitivity of commercially available assays for glucagon-like peptide-1 (GLP-1): implications for GLP-1 measurements in clinical studies. Diabetes Obes Metab.

[39] Runchey SS (2013). Effect of low- and high-glycemic load on circulating incretins in a randomized clinical trial. Metabolism.

[40] Ryskjaer J (2006). Plasma dipeptidyl peptidase-IV activity in patients with type-2 diabetes mellitus correlates positively with HbAlc levels, but is not acutely affected by food intake. Eur J Endocrinol.

[41] Pala L (2010). Relationship between GLP-1 levels and dipeptidyl peptidase-4 activity in different glucose tolerance conditions. Diabet Med.

[42] Carbone LD (2017). Association of DPP-4 activity with BMD, body composition, and incident hip fracture: the Cardiovascular Health Study. Osteoporos Int.

[43] Kim SW, Cho EH (2016). High Levels of Serum DPP-4 Activity Are Associated with Low Bone Mineral Density in Obese Postmenopausal Women. Endocrinol Metab (Seoul).

[44] Hansen L, Deacon CF, Orskov C, Holst JJ (1999). Glucagon-like peptide-1-(7-36)amide is transformed to glucagon-like peptide-1-(9-36)amide by dipeptidyl peptidase IV in the capillaries supplying the L cells of the porcine intestine. Endocrinology.

[45] Kyle KA, Willett TL, Baggio LL, Drucker DJ, Grynpas MD (2011). Differential effects of PPAR-{gamma} activation versus chemical or genetic reduction of DPP-4 activity on bone quality in mice. Endocrinology.

[46] Bohm SK, Gum JR, Erickson RH, Hicks JW (1995). & Kim, Y. S. Human dipeptidyl peptidase IV gene promoter: tissue-specific regulation from a TATA-less GC-rich sequence characteristic of a housekeeping gene promoter. Biochem J.

[47] Erickson RH (1999). Regulation of the gene for human dipeptidyl peptidase IV by hepatocyte nuclear factor 1 alpha. Biochem J.

[48] Mulvihill EE, Drucker DJ (2014). Pharmacology, physiology, and mechanisms of action of dipeptidyl peptidase-4 inhibitors. Endocr Rev.

[49] Bauvois B, Djavaheri-Mergny M, Rouillard D, Dumont J, Wietzerbin J (2000). Regulation of CD26/DPPIV gene expression by interferons and retinoic acid in tumor B cells. Oncogene.

[50] Cunha JS, Ferreira VM, Maquigussa E, Naves MA, Boim MA (2014). Effects of high glucose and high insulin concentrations on osteoblast function *in vitro*. Cell Tissue Res.

[51] Levinger, I. *et al*. Glucose-loading reduces bone remodeling in women and osteoblast function *in vitro*. *Physiol Rep* **4**, doi:10.14814/phy2.12700 (2016). 10.14814/phy2.12700 PMC4758933 26847728

[52] Marcil V (2015). Hepatocyte nuclear factor 4 alpha polymorphisms and the metabolic syndrome in French-Canadian youth. PLoS One.

[53] Wilcox G (2005). Insulin and insulin resistance. Clin Biochem Rev.

[54] Turcot V (2011). DPP4 gene DNA methylation in the omentum is associated with its gene expression and plasma lipid profile in severe obesity. Obesity (Silver Spring).

[55] Aertgeerts K (2004). N-linked glycosylation of dipeptidyl peptidase IV (CD26): effects on enzyme activity, homodimer formation, and adenosine deaminase binding. Protein Sci.

[56] Bilodeau N (2006). Insulin-dependent phosphorylation of DPP IV in liver. Evidence for a role of compartmentalized c-Src. FEBS J.

[57] Tremaroli V, Backhed F (2012). Functional interactions between the gut microbiota and host metabolism. Nature.

[58] Backhed F (2004). The gut microbiota as an environmental factor that regulates fat storage. Proc Natl Acad Sci USA.

[59] Ley RE (2005). Obesity alters gut microbial ecology. Proc Natl Acad Sci USA.

[60] Larsen N (2010). Gut microbiota in human adults with type 2 diabetes differs from non-diabetic adults. PLoS One.

[61] Reimer RA, McBurney MI (1996). Dietary fiber modulates intestinal proglucagon messenger ribonucleic acid and postprandial secretion of glucagon-like peptide-1 and insulin in rats. Endocrinology.

[62] Scholz-Ahrens KE, Schrezenmeir J (2007). Inulin and oligofructose and mineral metabolism: the evidence from animal trials. J Nutr.

[63] Johnson CD (2011). Addition of fructooligosaccharides and dried plum to soy-based diets reverses bone loss in the ovariectomized rat. Evid Based Complement Alternat Med.

[64] Sauer J, Richter KK, Pool-Zobel BL (2007). Physiological concentrations of butyrate favorably modulate genes of oxidative and metabolic stress in primary human colon cells. J Nutr Biochem.

[65] Newman JC, Verdin E (2014). beta-hydroxybutyrate: much more than a metabolite. Diabetes Res Clin Pract.

[66] Lin HV (2012). Butyrate and propionate protect against diet-induced obesity and regulate gut hormones via free fatty acid receptor 3-independent mechanisms. PLoS One.

[67] Edfalk S, Steneberg P, Edlund H (2008). Gpr40 is expressed in enteroendocrine cells and mediates free fatty acid stimulation of incretin secretion. Diabetes.

[68] Akoume MY (2010). Cell-based screening test for idiopathic scoliosis using cellular dielectric spectroscopy. Spine (Phila Pa 1976).

[69] Suzuki A, Nakauchi H, Taniguchi H (2003). Glucagon-like peptide 1 (1-37) converts intestinal epithelial cells into insulin-producing cells. Proc Natl Acad Sci USA.

[70] Mannucci E (2000). Glucagon-like peptide (GLP)-1 and leptin concentrations in obese patients with Type 2 diabetes mellitus. Diabet Med.

[71] Wang X, Zhou J, Doyle ME, Egan JM (2001). Glucagon-like peptide-1 causes pancreatic duodenal homeobox-1 protein translocation from the cytoplasm to the nucleus of pancreatic beta-cells by a cyclic adenosine monophosphate/protein kinase A-dependent mechanism. Endocrinology.

[72] Meng J (2016). Activation of GLP-1 Receptor Promotes Bone Marrow Stromal Cell Osteogenic Differentiation through beta-Catenin. Stem Cell Reports.

[73] Das SS (2014). Regulation of dipeptidyl peptidase 4 production in adipocytes by glucose. Diabetes Metab Syndr Obes.

[74] Moreau A (2004). Melatonin signaling dysfunction in adolescent idiopathic scoliosis. Spine (Phila Pa 1976).

[75] Marcil V (2010). Modification in oxidative stress, inflammation, and lipoprotein assembly in response to hepatocyte nuclear factor 4alpha knockdown in intestinal epithelial cells. J Biol Chem.

