# Supplementary material for: Dipeptidyl Peptidase-4 and Adolescent Idiopathic Scoliosis: Expression in Osteoblasts
Source: Sci Rep. 2017 Jun 9;7:3173. doi: 10.1038/s41598-017-03310-x (PMC5466660; doi:10.1038/s41598-017-03310-x)
Supplement: Supplementary file 1 — Supplementary data [file 41598_2017_3310_MOESM1_ESM.pdf]

Dipeptidyl Peptidase-4 and Adolescent Idiopathic Scoliosis: Expression in Osteoblasts

Emilie Normand[1,2], Anita Franco[1,3], Alain Moreau[3,4,5], Valérie Marcil[1,2*]

[1]Research Center of the Sainte-Justine University Hospital, Montreal, Quebec, H3T 1C5, Canada
[2]Department of Nutrition, Faculty of Medicine, Université de Montreal, Montreal, Quebec, H3T 1J4, Canada
[3]Viscogliosi Laboratory in Molecular Genetics of Musculoskeletal Diseases, Research Center of the Sainte-Justine University Hospital, Montreal, Quebec, H3T 1C5, Canada#1
[4]Department of Biochemistry and Molecular Medicine, Faculty of Medicine, Université de Montreal, Montreal, Quebec, H3T 1J4, Canada
[5]Department of Stomatology, Faculty of Dentistry, Université de Montréal, Montreal, Quebec, H3A 1J4, Canada

Supplementary Information

Supplementary Figure 1

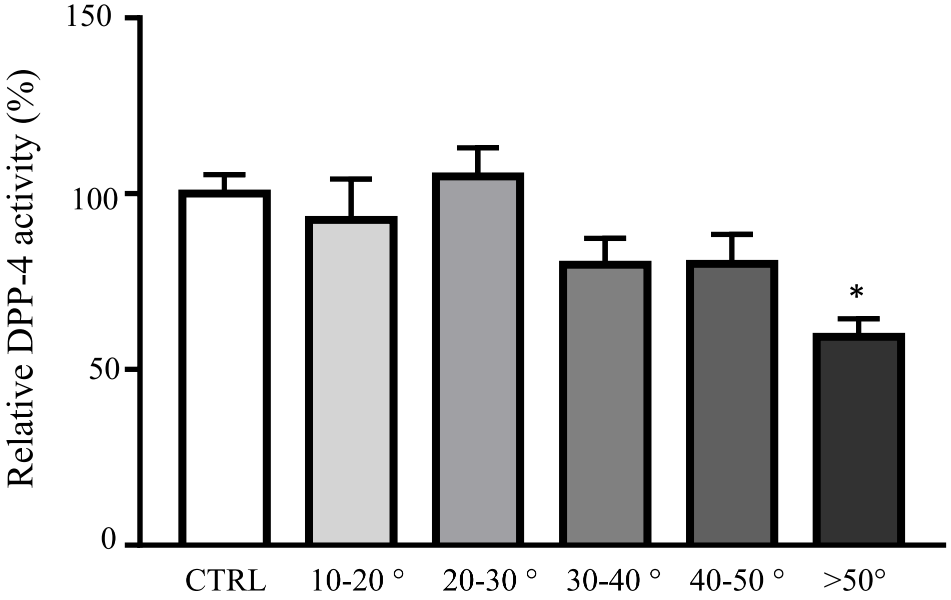

Supplementary Figure 2

a

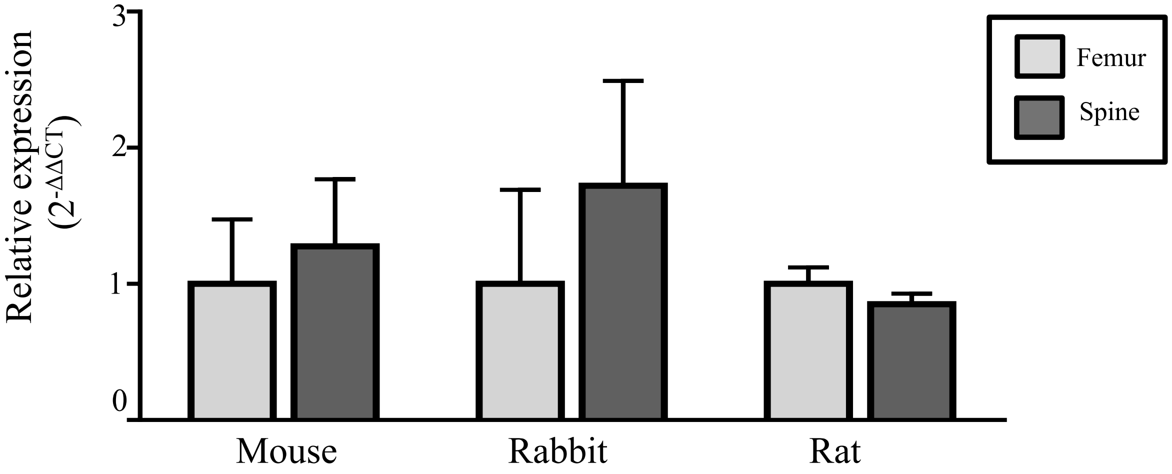

b

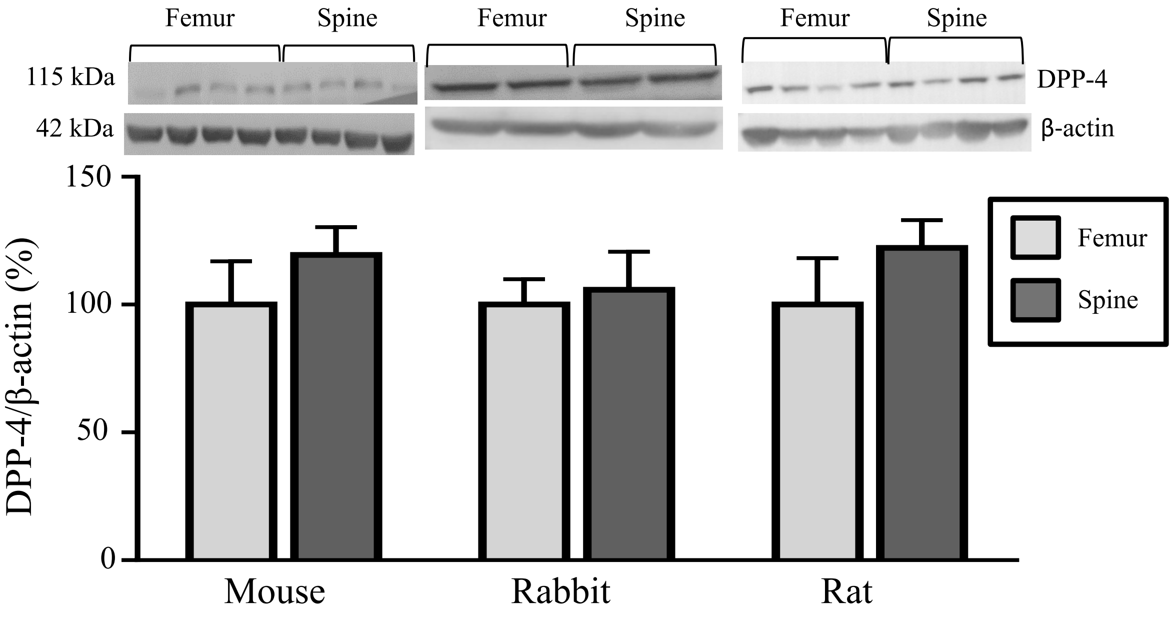

Supplementary Figure 3

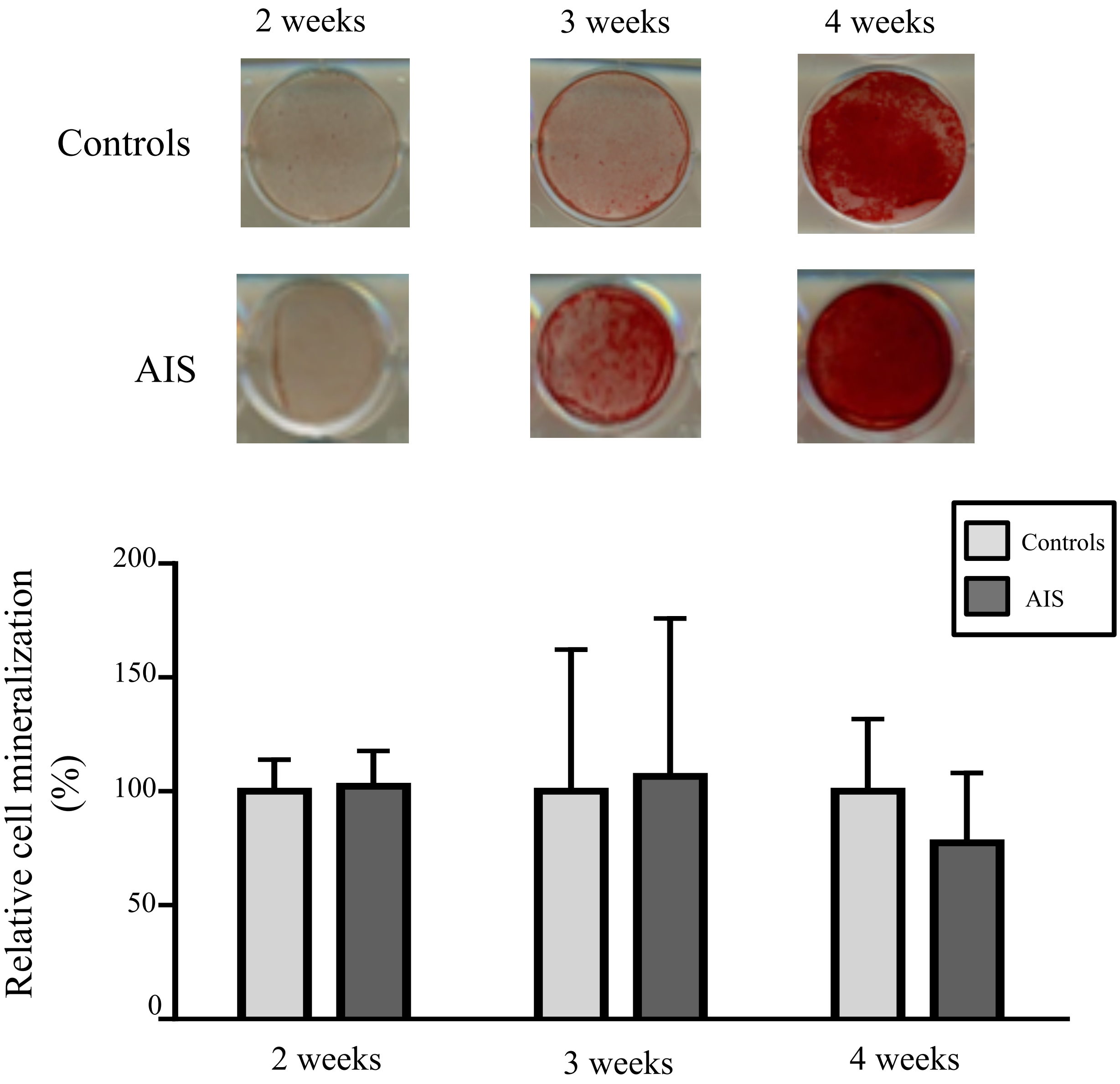

Supplementary Figure 4

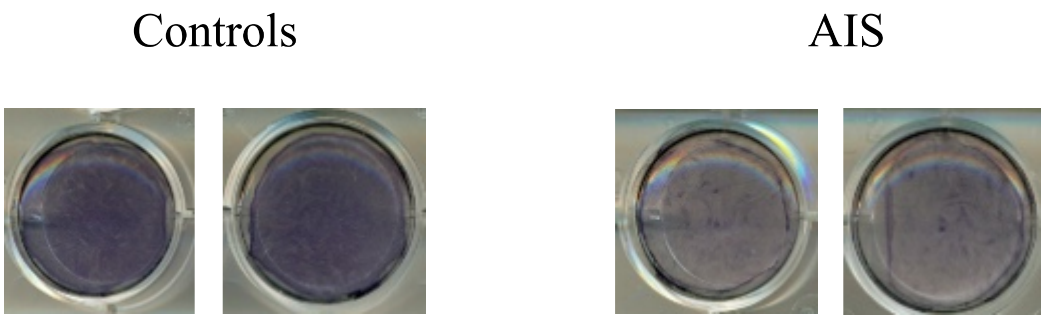

Supplementary Figure 5

a

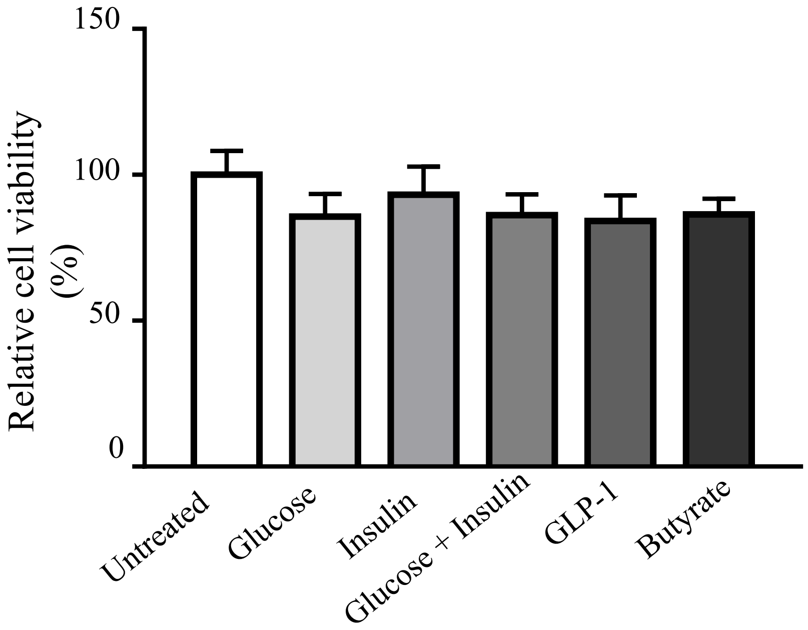

b

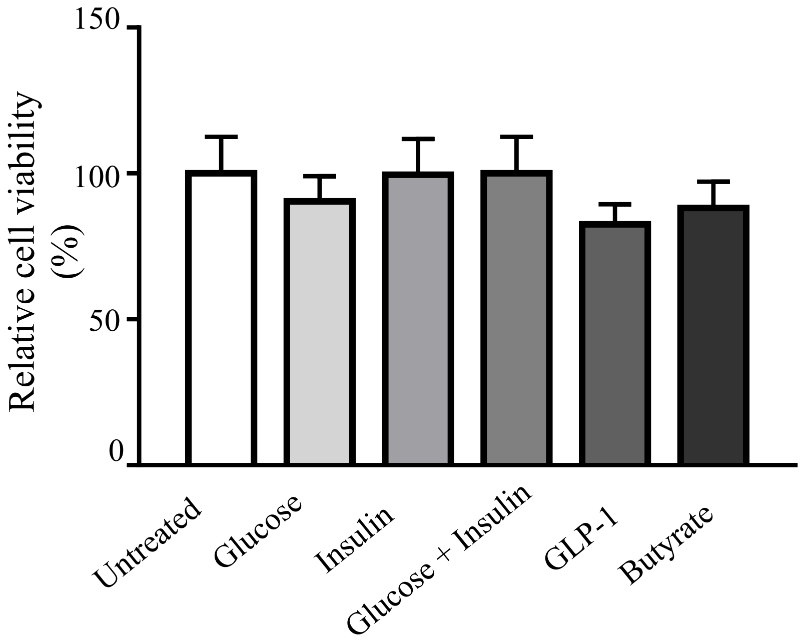

Supplementary Figure 6

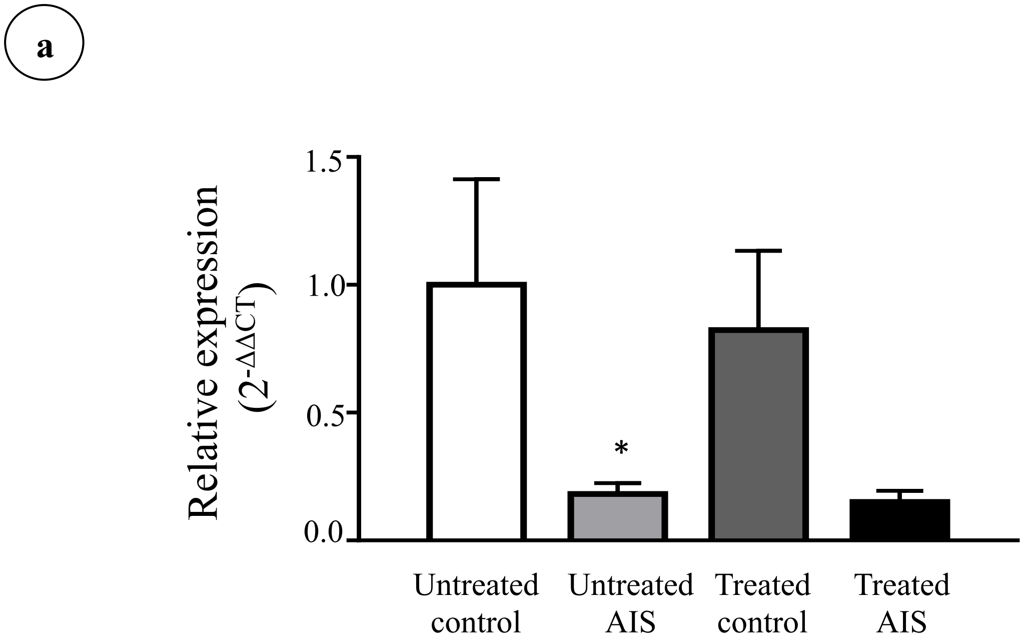

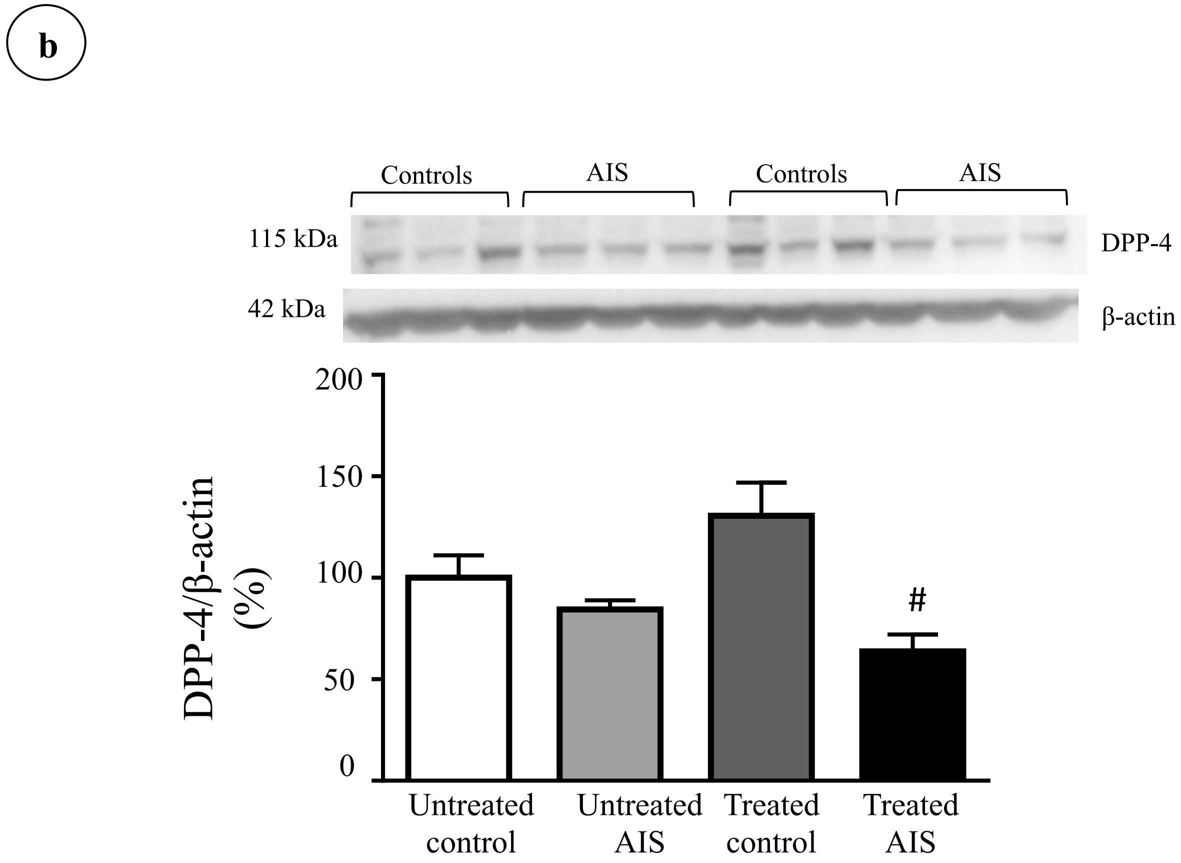

Supplementary Figure 7

a

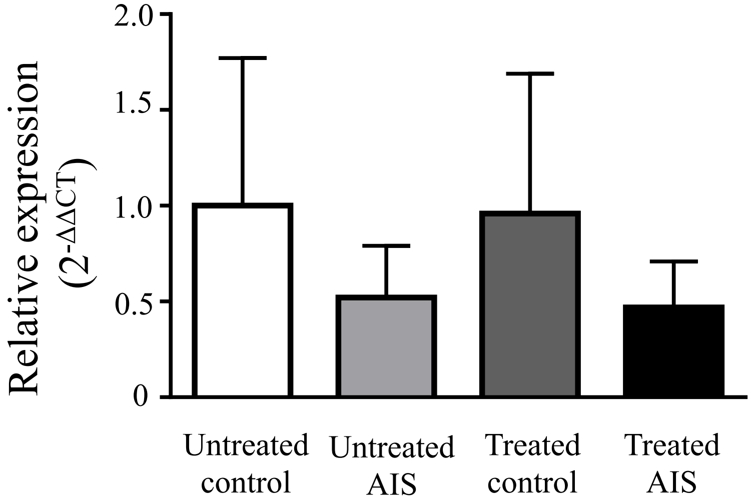

b

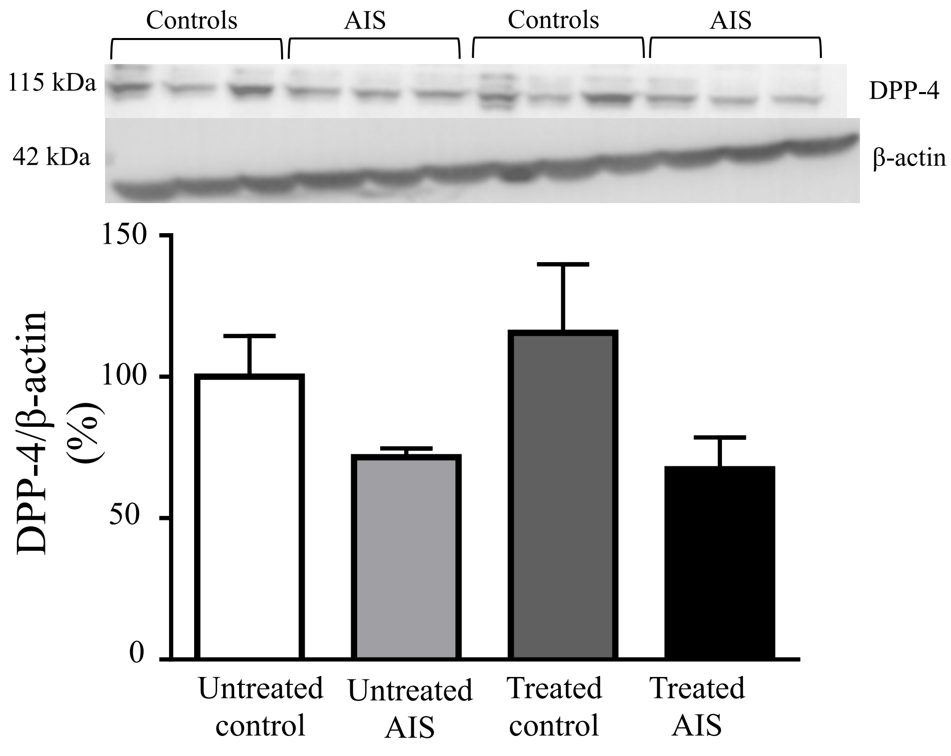

Supplementary Figure 8

a

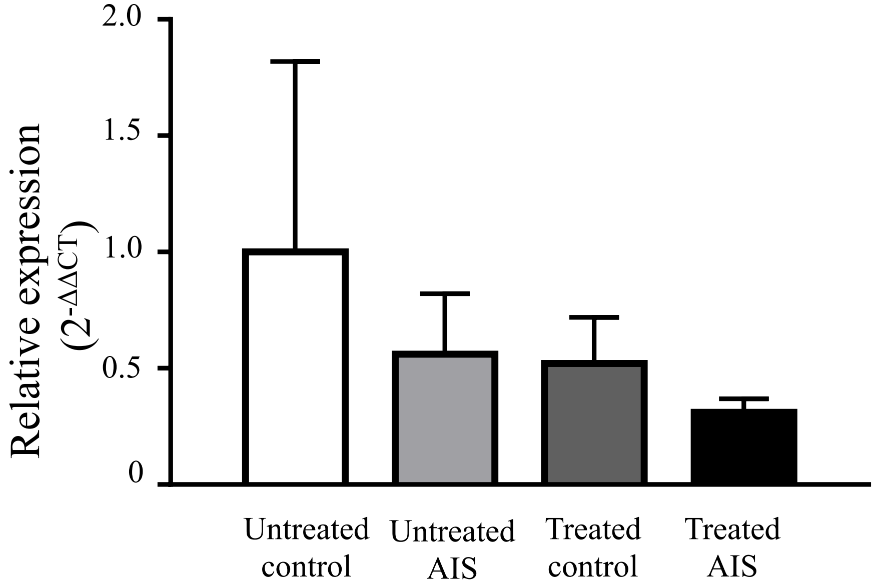

b

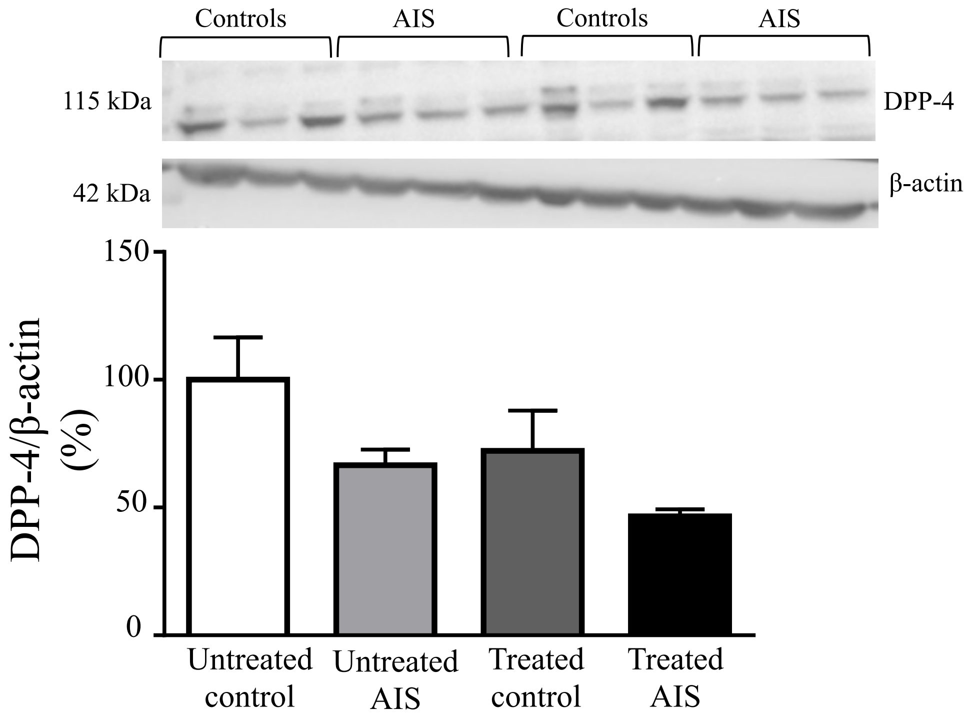

Supplementary Figure Legends

Supplementary Figure 1. Relative DPP-4 activity according to participants' highest Cobb angle. DPP-4 activity was measured using the DPPIV/CD26 Enzo Life Science's assay kit in plasma of 113 AIS girls and 62 age-matched controls. AIS participants were stratified according to highest Cobb angle: 10-20° (n=22), 21-30° (n=33), 31-40° (n=25), 41-50° (n=15) and over 50° (n=15). *P<0.05 vs. controls using one-way ANOVA followed by Tuckey's post-hoc tests. Data are presented as mean ± standard deviation.

Supplementary Figure 2. DPP-4 expression in osteoblasts from femurs and spines of mice, rabbits and rats. (a) Osteoblast *Dpp4* gene expression in femur and spine from mice (n=4), rabbits (n=2) and rats (n=5) was measured by RT-qPCR with *Gapdh* or *β-actin* as endogenous controls. Relative expression was analyzed with the $2^{-\Delta\Delta CT}$ method. (b) Osteoblast DPP-4 protein expression in femurs and spines from mice (n=4), rabbits (n=2) and rats (n= 4) measured by Western blot. β-actin was used as endogenous control. Two-tailed Student's t-tests were used for statistical analyses. Data are presented as mean ± standard deviation.

Supplementary Figure 3. Quantification of alizarin red staining of AIS and control osteoblasts. Quantification of alizarin red staining was performed via extraction with acetic acid. Dye was collected after 2 weeks (controls n=6; AIS n=5), 3 weeks (controls n=5; AIS n=5) and 4 weeks (controls n=6; AIS n=4) and quantified by spectrophotometry (405 nm). Statistical analyses were performed using one-way ANOVA. Representative pictures are shown. Data are presented as mean ± standard deviation.

Supplementary Figure 4. Alkaline phosphatase staining of controls and AIS osteoblasts. Representative pictures of controls and AIS osteoblasts after alkaline phosphatase staining using the BCIP/NBT liquid substrate system. The purple color validates the activity of the bone biomarker. Data are presented as mean ± standard deviation.

Supplementary Figure 5. Cell viability after 2 and 24 hours of treatment with effectors. Relative cell viability from AIS and control osteoblasts (n=5/group) was determined by MTT assay after treatments with glucose (5 mM), insulin (0.3 nM), glucose + insulin (5 mM and 0.3 nM), GLP-1 (10 nM) and butyrate (10 mM) for (a) 2 hours and (b) 24 hours. For each treatment, viability was evaluated relative to untreated cells. Statistical analyses were performed using one-way ANOVA. Data are presented as mean ± standard deviation.

Supplementary Figure 6. Impact of short-term treatments with GLP-1 on DPP-4 expression. Cells were treated with GLP-1 (10 nM) for 2 hours. (a) *DPP4* gene expression in osteoblasts of controls (n=3) and AIS patients (n= 6) was measured by RT-qPCR with *GAPDH* as endogenous control. Relative expression was analyzed with the $2^{-\Delta\Delta CT}$ method. (b) DPP-4 protein expression in osteoblasts of controls and AIS patients (n=3/group) was measured by Western blot with β-actin as endogenous control. *P<0.05 vs. untreated controls; $^{\#}$P<0.05 vs. treated controls using one-way ANOVA followed by Tuckey's post-hoc tests. Data are presented as mean ± standard deviation.

Supplementary Figure 7. Impact of short-term treatment with butyrate on DPP-4 expression. Cells were treated with butyrate (10 mM) for 2 hours. (a) *DPP4* gene expression in osteoblasts of controls (n=4) and AIS patients (n=5) was measured by RT-qPCR with *GAPDH* as

endogenous control. Relative expression was analyzed with the $2^{-\Delta\Delta CT}$ method. (b) DPP-4 protein expression in osteoblasts of controls and AIS patients (n=3/group) was measured by Western blot with β-actin as endogenous control. Statistical analyses were performed using one-way ANOVA. Data are presented as mean ± standard deviation.

Supplementary Figure 8. Impact of long-term treatment with butyrate on DPP-4 expression. Cells were treated with butyrate (10 mM) for 24 hours. (a) *DPP4* gene expression in osteoblasts of controls (n=4) and AIS patients (n=5) was measured by RT-qPCR with *GAPDH* as endogenous control. Relative expression was analyzed with the $2^{-\Delta\Delta CT}$ method. (b) DPP-4 protein expression in osteoblasts of controls and AIS patients (n=3/group) was measured by Western blot with β-actin as endogenous control. Statistical analyses were performed using one-way ANOVA. Data are presented as mean ± standard deviation.

Supplementary Table 1. Characteristics of control bone specimen donors for osteoblast cell culture

Group	Gender	Age (years)	Anatomical site
Control	F	15.1	Femur
Control	F	14.0	Tibia
Control	F	18.7	Tibia
Control	F	18.7	Tibia
Control	F	11.6	Tibia
Control	F	15.2	Tibia and femur
Control	F	15.5	Tibia and fibula
Control	M	16.6	Femur
Control	M	14.1	Femur
Control	M	13.8	Tibia
Control	M	12.1	Tibia
n=11	63.6% female	Average age: 15.0 ± 2.3	

Supplementary Table 2. Characteristics of AIS bone specimen donors for osteoblast cell culture.

Group	Gender	Age (years)	BMI (kg/m^2)	Risser score	Highest Cobb angle (°)	Curve type
AIS	F	11.3	18.7	n/a	54	rT-lL
AIS	F	13.3	21.0	3	47	rT-ITL
AIS	F	13.5	16.4	0	61	IT-rT-IL
AIS	F	14.1	21.6	n/a	52	rT
AIS	F	14.4	17.8	n/a	71	rT-lL
AIS	F	14.4	26.4	n/a	44	rT-lTL
AIS	F	14.8	18.7	n/a	47	lT-rT-lL
AIS	F	14.9	29.0	n/a	66	rT
AIS	F	15.3	23.5	n/a	45	rT-ITL
AIS	F	15.5	20.2	4	67	rT
AIS	F	16.7	22.9	n/a	56	rT-lL
AIS	F	16.9	22.3	n/a	52	rT
AIS	F	19.3	20.6	n/a	53	lT-rT-lL
n=13	100% female	Average ± SD: 15.0 ± 2.0	Average ± SD: 21.5 ± 3.5		Average ± SD: 55 ± 9	

All specimens were obtained from the spine (curve apex). BMI: Body mass index; AIS: Adolescent idiopathic scoliosis; Curve type: r: right; l; left; T: Thoracic; L: Lumbar; TL: Thoracolumbar; SD: standard deviation.
